# Wavefront-Guided Photorefractive Keratectomy with the Use of a New Hartmann-Shack Aberrometer in Patients with Myopia and Compound Myopic Astigmatism

**DOI:** 10.1155/2015/514837

**Published:** 2015-10-04

**Authors:** Steven C. Schallhorn, Jan A. Venter, Stephen J. Hannan, Keith A. Hettinger

**Affiliations:** ^1^University of California, San Francisco, 11730 Caminito Prenticia, San Diego, CA 92131, USA; ^2^Optical Express, 5 Deerdykes Road, Cumbernauld G68 9HF, UK; ^3^Optical Express, 9820 Willow Creek Road, Suite 260, San Diego, CA 92131, USA

## Abstract

*Purpose*. To assess refractive and visual outcomes and patient satisfaction of wavefront-guided photorefractive keratectomy (PRK) in eyes with myopia and compound myopic astigmatism, with the ablation profile derived from a new Hartmann-Shack aberrometer. *Methods*. In this retrospective study, 662 eyes that underwent wavefront-guided PRK with a treatment profile derived from a new generation Hartmann-Shack aberrometer (iDesign aberrometer, Abbott Medical Optics, Inc., Santa Ana, CA) were analyzed. The preoperative manifest sphere ranged from −0.25 to −10.75 D, and preoperative manifest cylinder was between 0.00 and −5.25 D. Refractive and visual outcomes, vector analysis of the change in refractive cylinder, and patient satisfaction were evaluated. *Results*. At 3 months, 91.1% of eyes had manifest spherical equivalent within 0.50 D. The percentage of eyes achieving uncorrected distance visual acuity 20/20 or better was 89.4% monocularly and 96.5% binocularly. The mean correction ratio of refractive cylinder was 1.02 ± 0.43, and the mean error of angle was 0.00 ± 14.86° at 3 months postoperatively. Self-reported scores for optical side effects, such as starburst, glare, halo, ghosting, and double vision, were low. *Conclusion*. The use of a new Hartmann-Shack aberrometer for wavefront-guided photorefractive keratectomy resulted in high predictability, efficacy, and patient satisfaction.

## 1. Introduction

Although laser in situ keratomileusis (LASIK) outperforms photorefractive keratectomy in early postoperative stages [1], there are patients where the choice of surface ablation over the flap creation is well justified [2, 3]. These include eyes with thinner corneas, superficial scars, and epithelial basement membrane dystrophy and eyes where ocular pathology might create concerns for increased intraocular pressure during femtosecond flap creation or simply patients who wish to preserve corneal integrity due to the increased risk of trauma in their profession.

Since the advent of PRK over two decades ago, several improvements were introduced to maximize the postoperative visual quality, one of them being the use of wavefront-guided ablation profiles. When it comes to the higher order aberrations treatment, some studies even suggest that PRK might offer an advantage over LASIK [4–6]. Corneal flap creation induces its own aberrations that are unaccounted for in preoperative planning, although this risk has significantly decreased with the introduction of femtosecond lasers [7]. Wavefront-guided ablation profiles as such undergo continuous improvements, with more sophisticated wavefront mapping devices being introduced. In this study, we evaluate the results of a large consecutive cohort of patients with myopia/myopic astigmatism undergoing the wavefront-guided surface ablation with the ablation profile derived from the new Hartmann-Shack aberrometer (iDesign Advanced Wavescan Studio, Abbott Medical Optics, Inc., Santa Ana, CA). Accuracy of refractive and astigmatic correction, visual acuities, and patient satisfaction, including self-reported night vision disturbances, are evaluated up to 3 months postoperatively.

## 2. Patients and Methods

This retrospective, noncomparative study was deemed exempt from full review by the Committee on Human Research at the University of California, San Francisco, because it used only retrospective, deidentified patient data. Informed consent to undergo PRK procedure was obtained from all patients.

The deidentified patients records were extracted from an electronic database with the following criteria: cases that underwent primary photorefractive keratectomy targeted for emmetropia and were available for 1 week, 1 month, and 3 months following operative examination; preoperative myopic refraction with less than 12.00 D of spherical myopia, less than 6.00 D of refractive astigmatism, and no more than 12.00 D of manifest spherical equivalent; surgeries performed with the Visx Star S4 IR excimer laser (Abbott Medical Optics, Santa Ana, CA) using a wavefront-guided ablation profile derived from a new diagnostic device (iDesign System); visual acuity correctable to 20/32 or better in both eyes and age of 18 years or older. Data extraction techniques have been previously described [8].

Exclusion criteria for treatment were active ophthalmic disease, abnormal corneal shape, concurrent medications or medical conditions that could impair healing, and the final calculated stromal bed thickness less than 350 *μ*m. Soft contact lens wearers were asked to discontinue use at least 7 days prior to the procedure. Hard contact lens users (PMMA or rigid gas permeable lenses) removed their lenses at least 3 weeks prior to baseline measurements and had two central keratometry readings and two manifest refraction values taken at least 1 week apart that did not differ by more than 0.50 D in either meridian.

Preoperative examination included manifest and cycloplegic refraction, monocular and binocular uncorrected distance visual acuity (UDVA), corrected distance visual acuity (CDVA) using a calibrated projected eye chart, low-light pupil diameter, slit-lamp biomicroscopy, dilated fundus examination, applanation tonometry, corneal topography, ultrasound pachymetry, and wavefront aberration measurement.

Postoperative examinations were conducted at 1 day, 4 days, 1 week, 1 month, and 3 months. On the first and fourth postoperative day, a detailed slit-lamp examination of the cornea was performed and unaided visual acuity checked. On the fourth day, the bandage contact lens was removed, if the process of reepithelization was completed. At the subsequent postoperative visits, the same preoperative examination protocol (excluding cycloplegic refraction, pupil diameter, topography, aberrometry, and pachymetry) was used. As part of current practice, all patients were asked to complete a questionnaire et each postoperative visit. The questionnaire from the last available visit was used for analysis. It was self-administered by the patient using a password protected and secure computer terminal in an isolated area of the clinic. The questionnaire responses were stored in the secured Optical Express central database, which is compliant with ISO 27001 for information security management systems. The questionnaire was derived from the Joint LASIK Study Task Force [9] (Table 1). All response fields utilized a Likert scale to obtain the patient's preferences or degree of agreement. Night vision phenomena such as starburst, glare, halo, ghosting, and double vision and difficulty with dry eyes were rated on the scale from 1 (no difficulty) to 7 (severe difficulty).

### 2.1. Surgical Technique

All PRK procedures were performed by experienced surgeons certified to use the equipment. The eye was first anaesthetized with topical proxymetacaine hydrochloride 0.5%, and a 9 mm well was placed on the cornea and filled with 20% ethanol. Following a 30–40 seconds' application, the alcohol was carefully drained with a surgical spear and the epithelium removed with a blunt spatula. Wavefront-guided excimer laser ablation was performed using the Visx Star S4 IR laser system (Abbott Medical Optics) with iris registration. The ablation algorithm was derived from the iDesign aberrometer. For all treatments, the optical zone diameter was 6.0 mm with a transition zone of 8.0 mm. In patients with astigmatism, 6.0 mm was the size of the minor axis of the elliptical ablation. A nomogram was used to adjust the sphere according to the magnitude of the aberrometer-derived cylinder to avoid overcorrection of sphere when treating high cylinder (Table 2).

For stromal ablations greater than 70 *μ*m, a circular sponge soaked in mitomycin C 0.02% was applied for 20 seconds. The ocular surface was then thoroughly rinsed with 15 mL of balanced salt solution. A bandage contact lens (PureVision, Bausch & Lomb) was placed on the eye and left in place until the cornea reepithelialized.

Postoperative medications consisted of topical levofloxacin 0.5%, 4 times a day for one week, and 4 weeks of tapering dose of topical fluorometholone ophthalmic solution 0.1% in the following sequence: 4 times a day for 1 week, 3 times a day for 1 week, 2 times a day for one week, and once a day for one week.

### 2.2. Wavefront Sensor

The iDesign System is a new generation high definition Hartmann-Shack aberrometer that has greater dynamic range (−16.0 to +12.0 D with up to +8.0 D of cylinder and up to 8 *μ*m of HOA RMS) and higher resolution than previous generation Hartmann-Shack devices. Refraction measured by this wavefront sensor was found to have high repeatability [10]. The data reconstruction is done by Fourier algorithms, using up to 1,257 data points over a 7 mm pupil diameter. The device performs five ocular measurements within a single capture sequence: topography, autorefractometry, pupillometry, and keratometry.

### 2.3. Statistical Analysis

Snellen visual acuity was converted into logMAR for statistical analysis, and all continuous variables were described with mean, standard deviation, and range. Paired Student's *t*-test was used for comparisons between consecutive visits. Correlation coefficients were calculated to assess the correlation between different variables. To evaluate the change in refractive cylinder, vector analysis was performed, using a previously described technique [11]. All data were analysed using Microsoft Office Excel 2007 program (Microsoft Corp.) and STATISTICA (StatSoft Inc.) on a personal computer. A level of significance of *P* = .05 was used.

## 3. Results

This study comprised 662 consecutive eyes of 352 patients that underwent primary myopic wavefront-guided photorefractive keratectomy between December 2013 and June 2014. Demographics of the study group, as well as the main preoperative and 3 months' postoperative outcomes, are summarized in Table 3.

### 3.1. Refractive and Visual Outcomes

**Figure 1:**
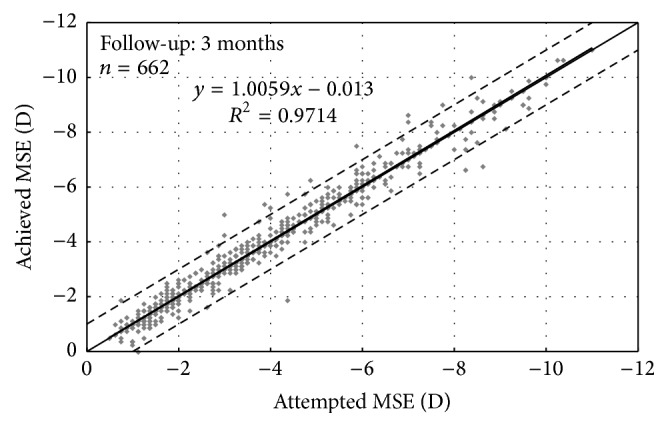
Predictability of manifest spherical equivalent (MSE). The area between two dotted lines is the postoperative MSE within ±1.00 D. The solid black line represents linear regression.

**Figure 2:**
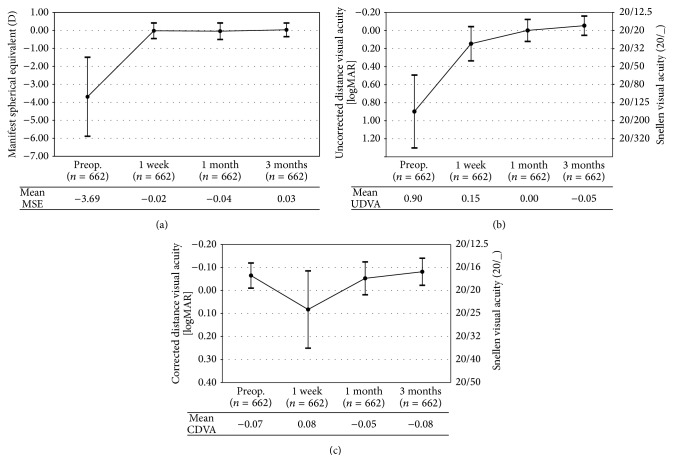
Change in refraction and visual acuities over time: (a) change in manifest spherical equivalent (MSE), (b) change in uncorrected distance visual acuity (UDVA), and (c) change in corrected distance visual acuity (CDVA). Error bars represent ± one standard deviation.

Figure 1 displays the predictability of the manifest spherical equivalent (MSE) at 3 months postoperatively. There was a strong and statistically significant correlation between the attempted and achieved MSE (*r* = 0.99, *P* < .01). The percentages of eyes with the 3 months' postoperative MSE within ±0.50 D and within ±1.00 D were 91.1% (603 eyes) and 97.6% (646 eyes), respectively. There was a statistically significant reduction in sphere, cylinder, and MSE postoperatively (Table 3).  Table 4 shows the stability of manifest refraction between consecutive visits. There was no statistically significant change in manifest sphere between 1-week and 1-month and between 1-month and 3-month examinations. Manifest cylinder reduced significantly between 1-month and 3-month visits (change by 0.19 D, *P* < .01), which resulted in a slight increase of MSE between 1-month and 3-month visits (change by +0.07 D, *P* < .01).  Figure 2 shows the change in MSE, UDVA, and CDVA over 3-month postoperative period.

**Figure 3:**
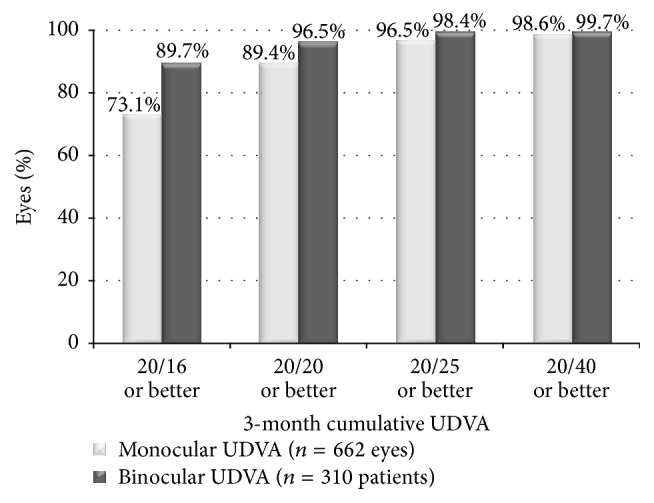
Cumulative monocular and binocular uncorrected distance visual acuity (UDVA) at 3 months postoperatively.

**Figure 4:**
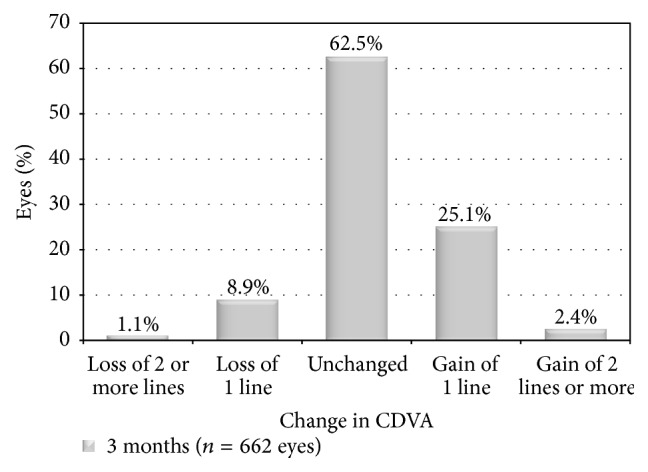
Safety comparison of preoperative and postoperative corrected distance visual acuity (CDVA) at 3 months postoperatively.

Figure 3 displays the 3 months' postoperative monocular and binocular cumulative UDVA. The percentage of eyes with monocular UDVA 20/20 or better was 89.4% (592 eyes). In patients that had both eyes treated, the percentage of patients achieving binocular UDVA 20/20 or better was 96.5% (299 patients).  Figure 4 depicts safety, the change between preoperative and postoperative CDVA. At 3 months postoperatively, 1.1% (7 eyes) lost 2 or more lines of CDVA, whereas 27.5% (182 eyes) gained 1 or more lines. The loss of 2 lines of CDVA was due to ocular surface issues, such as reduced tear break-up time and the presence of superficial punctate keratitis (4 eyes) and unresolved haze (3 eyes).

### 3.2. Vector Analyses of Refractive Astigmatism

**Figure 5:**
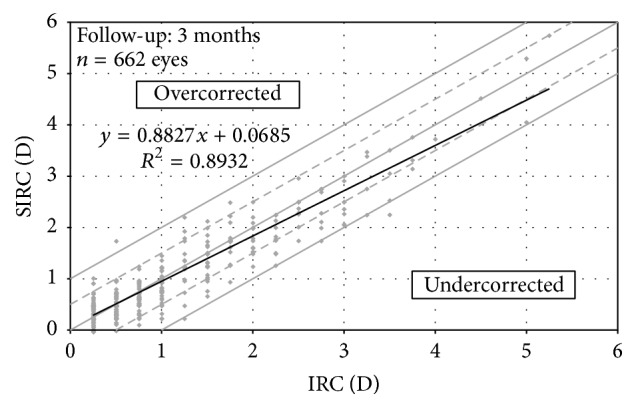
Intended refractive correction (IRC) versus surgically induced refractive correction (SIRC) at 3 months postoperatively. Solid black line is the linear regression.

Table 5 summarizes the mean values for vector analysis of the change in refractive astigmatism. At 3 months, 93.6% of eyes had a mean error of magnitude (difference between SIRC and IRC) within 0.50 D. The mean correction ratio (|SIRC|/|IRC|) was 1.02 ± 0.43, and the mean error of angle was 0.00 ± 14.86°.  Figure 5 plots the surgically induced refractive correction (SIRC) against intended refractive correction (IRC). Strong and statistically significant correlation was found between IRC and SIRC (*r* = 0.95, *P* < .01, Figure 5). The linear regression of IRC versus SIRC had a slope of 0.88 and intercept of 0.07 (Figure 5).

### 3.3. Patient Reported Outcomes

Out of 352 patients, 296 (84.1%) completed the postoperative patient satisfaction questionnaire with the mean follow-up of 3.1 ± 0.9 months. The percentage of patients willing to recommend the procedure to their friends and relatives was 95.5%. All scores for night vision disturbances had a median of 1 (no difficulty). A small percentage of patients (0.4%) claimed to have a lot of difficulty with night driving, and 0.4% felt unable to drive at night because of their vision. The percentage of patients being “dissatisfied” or “very dissatisfied” with their uncorrected vision was 2.3%. There was a statistically significant correlation between the magnitude of postoperative MSE, postoperative UDVA, and patients' satisfaction with visual outcomes (MSE versus satisfaction: *r* = 0.10, *P* = .01; UDVA versus satisfaction: *r* = 0.20, *P* < .01). Postoperative UDVA was also correlated to the scores for night vision disturbances and night driving (starburst: *r* = 0.08, *P* = .03; glare: *r* = 0.14, *P* < .01; halo: *r* = 0.12, *P* < .01; ghosting/double vision: *r* = 0.13, *P* < .01; night driving: *r* = 0.08, *P* = .03).

## 4. Discussion

Outcomes of wavefront-guided keratorefractive procedures, developed to target eye's preexisting lower and higher order aberrations, can be influenced by several factors. These include quality, resolution, and repeatability of the preoperative diagnostic equipment [12, 13], as well as the technology engaged in precise alignment of ablation profile on patient's cornea during the excimer laser procedure [14]. The new iDesign System has the highest resolution of any Hartmann-Shack aberrometer available in clinical practice. The device can capture up to 1257 data points, depending on pupil size, allowing more precise determination of true ocular wavefront and better accuracy of aberrometer-derived refraction, including magnitude and axis of astigmatism. Another feature of this new device is an enhanced iris registration system, which may improve rotational and directional alignment of the ablation profile. Correct alignment of ablation on patient's cornea is critical when the treatment of higher order aberrations is attempted. Wavefront-guided ablations tend to be more sensitive to any misalignment (whether caused by an initial placement error or by intraoperative eye movement) than standard spherocylindrical corrections [24, 25]. In this study, we analyzed the outcomes of wavefront-guided photorefractive keratotomy in a large cohort of consecutive patients with a new generation Hartmann-Shack aberrometer used in preoperative planning. To our knowledge, this is the first study evaluating the use of this new device in treatment planning of surface ablations.

In this study, we observed the mean MSE of +0.03 ± 0.38 D at 3 months postoperatively, which is an excellent result considering the wide range of preoperative spherical equivalents (−0.50 to −11.00 D). The percentage of eyes with postoperative MSE within 0.50 D and 1.00 D was 91.1% and 97.6%, respectively. At 3 months, 89.4% of eyes had monocular UDVA of 20/20 or better, and the percentage of patients achieving binocular UDVA 20/20 or better was 96.5%. The change in manifest sphere was relatively stable between 1-week and 1-month and between 1-month and 3-month visits. However, manifest cylinder improved significantly between the 1-month and 3-month postoperative examinations (Table 4).  Table 6 presents a summary of the literature [5, 6, 15–23] with refractive and visual outcomes of wavefront-guided PRK using different laser platforms and aberrometers. Most of the studies were performed with a small number of subjects, which makes the comparison difficult. Despite this, our results are comparable or superior to the studies in this review.

When it comes to the astigmatic correction, we achieved a mean correction ratio (ratio of the magnitude of SIRC to IRC) of 1.02 ± 0.43 at 3 months, which is comparable to the previous report on the use of the same aberrometer (iDesign System) in eyes undergoing wavefront-guided LASIK (1.02 ± 0.30, *n* = 243) [23]. The mean error of magnitude was +0.04 ± 0.27 D (arithmetic difference of the magnitudes between SIRC and IRC), and the mean error of angle was 0.00 ± 14.86° (angular difference between attempted treatment and achieved treatment). Hardly did any studies of wavefront-guided surface ablation report detailed vector analysis of astigmatic correction; however, Table 6 shows the magnitude of residual cylinder in each study found in the literature [5, 6, 15–23]. We achieved the lowest magnitude of postoperative cylinder (0.22 D) and one of the lowest standard deviations (0.28) despite having the largest cohort of patients and the widest range cylindrical correction (0 to 5.25 D). In addition, the scattergram of IRC versus SIRC shows undercorrection for higher values of preoperative cylinder (linear regression slope of 0.88, Figure 5), which could potentially be further improved with nomogram refinement.

One of the main reasons for considering the use of WFG ablation is its ability to minimize the induction of higher order aberrations (HOAs). The induction of HOA typically observed when using standard spherocylindrical corrections can result in unwanted optical side effects, as well as reduced patient satisfaction. The postoperative satisfaction with visual outcomes in our study was correlated to the amount of residual refractive error. We measured scores for night vision disturbances on the scale from 1 (no difficulty) to 7 (severe difficulty) and achieved mean scores of 1.55 ± 0.99 for starburst, 1.57 ± 0.95 for glare, 1.50 ± 0.92 for halos, and 1.38 ± 0.89 for ghosting and double vision at the 3.1 ± 0.9 months' follow-up visits. A prospective study by He and Manche [26] reports postoperative scores for night vision phenomena for WFG PRK, using a scale from 0 (no symptoms) to 10 (disabling symptoms) as follows: between 1 and 1.5 for night time glare and halos and close to 0.5 at 12 months postoperatively for diplopia/ghosting. Another study [18] found similar scores (1 to 1.5 for glare and halos at 12 months) measured on the same scale from 0 to 10, with the highest peak of symptoms at 1 month postoperatively (both night time glare and halos close to 2.5).

We found favorable outcomes with wavefront-guided PRK with ablation profiles derived from the new generation Hartmann-Shack aberrometer. Although LASIK is the preferred surgical choice in our practice, there are still a reasonable number of patients where surface ablation is selected, following surgeon/patient discussion. Many studies confirmed that the two techniques have similar efficacy, predictability, and safety, with the important exception of the slower visual recovery for PRK [1]. Some studies even found that PRK might induce fewer higher order aberrations compared to LASIK. Review of the literature regarding the impact of flap creation on postoperative higher order aberrations is mixed. For example, the study of Barreto Jr. et al. [27] found no difference in postoperative HOA between WFG LASIK and PRK, as far as the microkeratome flap creation is concerned, whereas other studies [4, 6] found fewer HOAs being induced with the surface ablation technique. Femtosecond flap is known to have minimal impact on HOA [7]. However, a study by Moshirfar et al. [5] found statistically significantly higher induction of HOAs with femtosecond LASIK (increase factor 1.74) than with PRK (increase factor 1.22), with wavefront-guided ablation used in both cases.

Our study has several limitations. Firstly, it is retrospective, and no comparison of preoperative and postoperative higher order aberrations was conducted. Secondly, refractive outcomes are evaluated over a 3-month period; long-term data might be necessary to confirm refractive stability. Despite the limitations, results of a large consecutive cohort of patients are presented in this study. The use of a new generation Hartmann-Shack aberrometer resulted in high predictability, efficacy, and safety, in a wide range of refractive errors undergoing a surface ablation treatment. Preoperative to postoperative changes in higher order aberrations should be further investigated.

## Figures and Tables

**Table 1:** Patient satisfaction questionnaire (follow-up: 3.1 ± 0.9 months, *n* = 296 patients).

Questions	Responses
Thinking about your vision during the last week, how satisfied are you with your vision? (without the use of glasses or contact lenses)	
Very satisfied	58.1%
Satisfied	36.7%
Neither	2.9%
Dissatisfied	1.8%
Very dissatisfied	0.5%
Because of your eyesight, how much difficulty do you have driving at night?	
No difficulty at all	61.0%
A little difficulty	23.6%
Moderate difficulty	5.9%
A lot of difficulty	0.4%
I am unable to drive at night because of my vision	0.4%
I do not drive at night for other reasons	8.8%
Would you recommend vision correction surgery to your friends and relatives?	
Yes	95.5%
No	4.5%

Visual phenomena	Mean ± SD (median)

Night vision phenomena scores measured on scale from 1 (no difficulty) to 7 (severe difficulty)	
Starburst	1.55 ± 0.99 (1)
Glare	1.57 ± 0.95 (1)
Halo	1.50 ± 0.92 (1)
Ghosting/double vision	1.38 ± 0.89 (1)
Dry eye symptoms rated on scan from 1 (no difficulty) to 7 (severe difficulty)	
Dry eye score (mean ± SD (median))	2.17 ± 1.31 (2)

**Table 2:** Nomogram for physician adjustment based on preoperative cylinder values obtained with the aberrometer. D: diopters.

Preoperative cylinder on aberrometry (D) (range)	Physician adjustment for sphere (D)
0 to 0.25	−0.25
0.26 to 0.75	−0.13
0.76 to 1.00	0.00
1.01 to 2.00	0.20
2.01 to 3.00	0.40
3.01 to 4.00	0.60
4.01 to 5.00	0.80
5.01 to 6.00	1.00
6.01 to 7.00	1.20
7.01 to 8.00	1.40

**Table 3:** Demographics and preoperative and 3 months' postoperative outcomes (*n* = 662 eyes).

	Preoperative	3 months' postoperative	*P* value
Age (years)			
Mean ± SD	32.3 ± 9.4	—	—
Range	(18 to 62)		
Gender			
Male/female	54%/46%	—	—
Eye			
Right/left	51%/49%	—	—
Sphere [D]			
Mean ± SD	−3.26 ± 2.18	+0.14 ± 0.38	<.01
Range	(−10.75 to −0.25)	(−1.75 to +2.25)	
Cylinder [D]			
Mean ± SD	−0.86 ± 0.83	−0.22 ± 0.28	<.01
Range	(−5.25 to 0.00)	(−2.00 to 0.00)	
MSE [D]			
Mean ± SD	−3.69 ± 2.20	+0.03 ± 0.38	<.01
Range	(−11.00 to −0.50)	(−2.50 to +2.00)	
UDVA [logMAR]			
Mean ± SD	0.90 ± 0.40	−0.05 ± 0.11	<.01
Range	(0.10 to 1.60)	(−0.20 to 0.60)	
CDVA [logMAR]			
Mean ± SD	−0.07 ± 0.05	−0.08 ± 0.06	<.01
Range	(−0.20 to 0.22)	(−0.20 to 0.40)	

**Table 4:** Stability of refraction (*n* = 662).

	1 week to 1 month	1 month to 3 months
Change in sphere by ≤0.5	82.9% (549 eyes)	92.1% (610 eyes)
Change in sphere by ≤1.0 D	95.0% (629 eyes)	98.9% (655 eyes)
Mean change in sphere ± SD	−0.01 ± 0.53	−0.02 ± 0.35
(*P* value)	(*P* = .50)	(*P* = .16)
95% CI	−0.05 to 0.03	−0.05 to 0.01

Change in Cyl by ≤0.5 D	74.9% (496 eyes)	87.0% (576 eyes)
Change in Cyl by ≤1.0 D	93.5% (619 eyes)	98.8% (654 eyes)
Mean change in Cyl ± SD	−0.02 ± 0.66^*∗*^	+0.19 ± 0.38^*∗*^
(*P* value)	(*P* = .48)	(*P* < .01)
95% CI	−0.07 to 0.03	0.16 to 0.22

Change in MSE by ≤0.5 D	82.8% (548 eyes)	89.3% (591 eyes)
Change in MSE by ≤1.0 D	96.7% (640 eyes)	99.1% (656 eyes)
Mean change in MSE ± SD	−0.02 ± 0.48	+0.07 ± 0.34
(*P* value)	(*P* = .35)	(*P* < .01)
95% CI	−0.06 to 0.02	0.04 to 0.10

MSE: manifest spherical equivalent; D: diopter; SD: standard deviation; CI: confidence interval; Cyl: cylinder.

^*∗*^Cylinder recorded in “minus” form. Positive change means reduction in manifest cylinder.

**Table 5:** Vector analysis of refractive astigmatism (follow-up: 3 months, *n* = 662).

Vector parameter	Mean ± SD (range)
Intended refractive correction [D]	0.86 ± 0.83 (0 to 5.25)
Surgically induced refractive correction [D]	0.92 ± 0.76 (0 to 5.71)
Error vector [D]	0.23 ± 0.29 (0 to 2.00)
Correction ratio	1.02 ± 0.43 (0 to 4.12)
Error of magnitude [D]	0.04 ± 0.27 (−1.25 to 1.25)
Error of angle [°]	0.00 ± 14.86 (−85.15 to 84.03)
Absolute error of angle [°]	4.97 ± 14.00 (0 to 84.03)
Axis shift [°]	0.13 ± 28.52 (−89.00 to 89.00)

SD: standard deviation; D: diopters.

**Table 6:** Summary of published results of wavefront-guided photorefractive keratectomy.

Author (year)	Number of eyes	Follow-up (months)	Laser	Preoperative		Postoperative		Postoperative MSE within		Postop. UDVA 20/20 or better	Loss of 2 or more lines of CDVA
				MSE [D] Mean ± SD (range)	Magnitude of cylinder [D] Mean ± SD (range)	MSE [D] Mean ± SD (range)	Magnitude of cylinder [D] Mean ± SD (range)	±0.50 D	±1.00 D		
Vinciguerra et al. (2007) [15]	68	3	NIDEK EC-5000 CX Il	−5.73 ± 2.03 (−2.50 to −11.25)	0.66 ± 0.59 (0 to 2.50)	−0.02 ± 0.78 (—)	—	92%	98%	100%	0%
Durrie et al. (2008) [16]	50	6	Alcon LADARVision 4000	−3.99 (—)	0.63 (0 to 2.75)	+0.08 (—)	0.26 (—)	—	—	94%	—
Karimian et al. (2010) [17]	28	8.1 ± 3.3	Technolas 217z	−4.92 ± 1.60 (−1.13 to −7.87)	0.93 ± 1.10 (—)	+0.19 ± 0.60 (−0.63 to +1.38)	0.57 ± 0.40 (0 to 1.50)	60.7%	100%	67.9%	—
Moshirfar et al. (2010) [5]	101	6	Visx Star S4 IR	−4.31 ± 2.01 (−1.53 to −8.07)	0.96 ± 0.78 (0 to 3.00)	+0.08 ± 0.35 (−0.75 to 1.50)	0.36 ± 0.37 (0 to 1.25)	77%	—	75%	2%
Manche and Haw (2011) [18]	34	12	Visx Star S4 IR	−4.39 ± 2.02 (−0.75 to −8.00)	0.85 ± 0.62 (0.0 to 2.50)	−0.17 ± 0.41 (—)	0.25 ± 0.25 (—)	91%	97%	97%	0%
Joosse et al. (2011) [19]	60	12	Technolas 217z	−6.05 ± 0.77 (−4.25 to −7.63)	0.88 ± 0.56 (0 to 2.25)	−0.02 ± 0.47 (−1.50 to 0.88)	0.32 ± 0.32 (0 to 1.25)	80%	96.7%	80%	6.7%
van Philips (2011) [20]	27	10	Technolas 217z100	−5.72 ± 0.88 (−4.25 to −7.50)	0.84 ± 0.59 ^*∗*^(0 to 2.25)	−0.03 ± 0.42 (−0.75 to +1.00)	0.32 ± 0.33 (0 to 1.25)	82%	100%	78%	0%
Bababeygy and Manche (2011) [21]	146	12	Visx Star S4 IR	−5.70 ± 2.54 (−0.13 to −10.30)	0.96 ± 0.81 (0 to 3.25)	−0.26 ± 0.31 (—)	0.30 ± 0.35 (0 to 1.50)	81.5%	96.6%	—	0%
Moshirfar et al. (2011) [22]	23	3	Visx Star S4 IR	−3.34 ± 1.75 (+1.0 to −8.50)	0.47 ± 0.35 (0 to 2.75)	+0.14 ± 0.31 (−0.38 to +0.88)	0.27 ± 0.25 (0 to 0.75)	96%	100%	91%	0%
Mifflin et al. (2012) [23]	40	12	Visx Star S4 IR	−3.22 ± 1.86 (+0.38 to −7.13)	0.72 ± 0.64 (0 to 2.50)	−0.08 ± 0.35 (−0.63 to +0.75)	0.31 ± 0.40 (0 to 1.25)	88.6%	94.3%	88.6%	0%
Ryan and O'Keefe (2012) [6]	38	12	Technolas 217z100	−3.99 ± 1.26 (−2.04 to −6.52)	1.01 ± 1.23 (−0.25 to 5.80)	−0.26 ± 0.31 (—)	0.47 ± 0.51 (—)	89.2%	97.3%	87%	0%
Current study	662	3	Visx Star S4 IR	−3.69 ± 2.20 (−0.50 to −11.00)	0.86 ± 0.83 (0 to 5.25)	+0.03 ± 0.38 (−2.50 to +2.00)	0.22 ± 0.28 (0 to 2.00)	91.1%	97.6%	89.4%	1.1%

^*∗*^Cylinder change in the table: positive change in cylinder means reduction of cylinder.
